# Functional Division Between the RecA1 and RecA2 Proteins in *Myxococcus xanthus*

**DOI:** 10.3389/fmicb.2020.00140

**Published:** 2020-02-12

**Authors:** Duo-Hong Sheng, Yi-Xue Wang, Miao Qiu, Jin-Yi Zhao, Xin-Jing Yue, Yue-Zhong Li

**Affiliations:** State Key Laboratory of Microbial Technology, Institute of Microbial Technology, Shandong University, Qingdao, China

**Keywords:** RecA, duplicate genes, *Myxococcus xanthus*, DNA recombination, antioxidation, functional divergence, SOS response

## Abstract

*Myxococcus xanthus* DK1622 has two RecA genes, *recA1* (MXAN_1441) and *recA2* (MXAN_1388), with unknown functional differentiation. Herein, we showed that both *recA* genes were induced by ultraviolet (UV) irradiation but that the induction of *recA1* was more delayed than that of *recA2*. Deletion of *recA1* did not affect the growth but significantly decreased the UV-radiation survival, homologous recombination (HR) ability, and induction of LexA-dependent SOS genes. In contrast, the deletion of *recA2* markedly prolonged the lag phase of bacterial growth and increased the sensitivity to DNA damage caused by hydrogen peroxide but did not change the UV-radiation resistance or SOS gene inducibility. Protein activity analysis demonstrated that RecA1, but not RecA2, catalyzed DNA strand exchange (DSE) and LexA autocleavage *in vitro*. Transcriptomic analysis indicated that RecA2 has evolved mainly to regulate gene expression for cellular transportation and antioxidation. This is the first report of functional divergence of duplicated bacterial *recA* genes. The results highlight the evolutionary strategy of *M. xanthus* cells for DNA HR and genome sophistication.

## Introduction

RecA, an ATP-dependent recombinase, is the core enzyme for DNA homologous recombination (HR), as well as being a promotion agent for LexA autolysis in bacteria (Lusetti and Cox, 2002). RecA also contributes to the repair of stalled and collapsed DNA replication forks by postreplication repair pathways (translesion DNA synthesis or template switching), playing an important role in DNA lesion tolerance pathways (Bichara et al., 2011; Quinet et al., 2017; Prado, 2018; Jaszczur et al., 2019). In addition, RecA participates in horizontal gene transfer between different strains (Lawrence and Retchless, 2009; Herrero-Fresno et al., 2015; García-Solache et al., 2016; He et al., 2016), which also causes genetic diversity. Thus, HR delicately balances genomic stability and diversity (Carr and Lambert, 2013; Greene, 2016). After binding to ssDNA, the RecA/ssDNA filament complex may serve as a signal of DNA damage, resulting in the self-cleavage of LexA, which activates the SOS response, increasing the expression of LexA-repressed genes. In the best characterized *Escherichia coli* SOS response, LexA autolysis derepresses the expression of more than 40 genes involved in DNA repair, mutagenesis, and many other cellular processes (Cox, 2003, 2007; Maslowska et al., 2019). Because of its pros and cons in genomic stability and variability, the functions of RecA are strictly regulated; for example, the function of RecA in *E. coli* is regulated at the gene transcription and protein activity levels. In the gene transcription induced by the SOS response, particularly, there is a 10–20 times difference in gene expression before and after induction (Cox, 1999, 2007).

Most bacteria, including *E. coli*, have a single *recA* gene, while some bacteria possess duplicate *recA* genes; however, duplicate *recA* genes have been investigated only in *Bacillus megaterium* and *Myxococcus xanthus*. In *B. megaterium*, duplicate *recA* genes were found to both be damage-inducible and similarly showed some DNA repair ability in *E. coli* (Nahrstedt et al., 2005). In the model strain of myxobacteria, *M. xanthus* DK1622, both RecA1 (MXAN_1441) and RecA2 (MXAN_1388) can partly restore the UV resistance of the *E. coli recA* mutant, and *recA2*, but not *recA1*, was found to be inducible by mitomycin or nalidixic acid (Norioka et al., 1995; Campoy et al., 2003). It is unclear how the duplicate RecA proteins play divergent functions in the DNA recombination and SOS induction in this organism.

In this study, we genetically and biochemically investigated the functions of RecA1 and RecA2 in *M. xanthus*. We found that both *recA* genes were inducible by UV irradiation but in different periods. The *recA1* deletion had no significant effects on cellular growth but reduced the UV-radiation resistance and induction ability of the SOS gene. In contrast, the absence of *recA2* did not affect irradiation resistance but significantly reduced bacterial growth and resistance to oxidative damage. *In vitro* protein activity analysis indicated that RecA1, but not RecA2, had the homologous strand exchange activity and was able to promote LexA autolysis. Transcriptomic analysis indicated that the *recA2* gene was crucial for intracellular substance transport and antioxidant capacity. We discuss the molecular mechanisms for the functional divergence of the RecA1 and RecA2 proteins.

## Materials and Methods

### Strains, Media, and DNA Substrates

The bacterial strains and plasmids used in this study are described in Supplementary Table S1. The *E. coli* strains were routinely grown on Luria-Bertani (LB) agar or in LB liquid broth at 37°C. The *M. xanthus* strains were grown in CYE liquid medium with shaking at 200 rpm or grown on agar plates with 1.5% agar at 30°C (Bretscher and Kaiser, 1978). When required, a final concentration of 40 μg/ml kanamycin (Kan) or 100 μg/ml ampicillin (Amp) was added to the solid or liquid medium.

Single-stranded viral DNA was isolated from M13mp18, and its 3 kb linear dsDNA was amplified by PCR and purified by a DNA purification kit (Tiangen, Beijing, China). A 60-nt oligomer from the M13 genome, 5′-CTG TCA ATG CTG GCG GCG GCT CTG GTG GTG GTT CTG GTG GCG GCT CTG AGG GTG GTG GCT-3′, was obtained from Tsingke Biotech (Qingdao). The 60-nt oligomer was ^32^P-labelled using a polynucleotide kinase (Ausubel et al., 1995) and stored in TE buffer (10 mM Tris–HCl, pH 7.0, and 0.5 mM EDTA).

### Growth and Resistance Analysis

*Myxococcus xanthus* strains were grown in CYE medium with shaking at 200 rpm at 30°C to an optical density of 0.5 at 600 nm (OD_600_). Cells were then collected by centrifugation at 8000 rpm for 10 min, washed with 10 mM phosphate buffer (pH 7.0), and diluted to 1 OD_600_ in the same buffer.

For the radiation damage assay, cells in 10 mM phosphate buffer (pH 7.0) were irradiated at room temperature with a gradient dose from 0 to 200 J/m^2^ using a UV Crosslinker (Fisher Scientific). Then, the cells were resuspended in fresh CYE medium and incubated at 30°C for 4 h. After incubation, cells were harvested by centrifugation and either used for a further assay or stored at −80°C.

For the oxidative damage assay, cells were suspended in phosphate buffer (pH 7.0) with a concentration of 1 OD, and hydrogen peroxide (H_2_O_2_) was added to a final concentration from 1 to 5 mM. The bacterial suspension was incubated for 20 min at room temperature with gentle shaking. After treatment, the suspension was immediately 10-fold diluted in the same phosphate buffer to end the oxidative damage reaction. Then, cells in the suspension were collected for further assay.

The growth assay was determined by growing cells in liquid medium at 30°C. Strains were inoculated at 0.02 OD_600_ and grown in CYE media for 84 h with shaking at 200 rpm. The OD_600_ was read every 12 h.

The survival rate was determined by a soft agar colony formation assay. Briefly, to determine the cell survival rate, *M. xanthus* cells were grown to the early exponential growth stage (OD ≈ 0.5). The cells were treated with UV or H_2_O_2_ as described above and were then diluted with fresh medium and mixed at a 1:2 ratio with melted 0.6% soft agar (50°C). The mixture was then spread on CYE plates. After a few minutes for medium solidification, the cultures were incubated at 30°C until clone formation. The survival percentage was calculated as the number of colony-forming units (CFUs) (damaged) divided by the total number of CFUs (undamaged).

### Homologous Recombination Assay

According to a previously reported method (Sheng et al., 2005), the recombination rate in *M. xanthus* was determined by measuring the probability of a resistance gene inserted into the genome through HR. The selected insertion site was located in the noncoding sequence between the MXAN_4466 and MXAN_4467 genes. Then, 500-bp fragments upstream and downstream of the insertion site were amplified with primers (UpF 5′-cacgggctacacgcaggtgcgggg-3′/UpR 5′-ttaagctttcgtttcagcggggactgcctgg-3′ and DownF 5′-caaagcttccaggcagtccccgctgaaacga-3′/DownR 5′-ggcatcgtccctggcggcggcgtgg-3′). The Kan resistance gene (*kanR*) with its promoter was simultaneously amplified from plasmid pZJY41 with primers 5′-gctgaagcttgtgctgaccccgggtgaat-3′/5′-agaagcttccagagtcccgctcagaagaac-3′. Then, the three DNA segments were linked by the *Hind*III site according to the arrangement of the upstream segment, resistance gene, and downstream segment. The linked DNA fragment was amplified with primers (UpF 5′-cacgggctacacgcaggtgcgggg-3′ and DownR 5′-ggcatcgtccctggcggcggcgtgg-3′) and quantitatively introduced into *M. xanthus* via electroporation (1.25 kV, 300 W, 50 mF, and 1 mm cuvette gap). A serial dilution was spread on CYE plates with or without Kan and incubated at 32°C for 72 h to count CFUs. The recombination ability was calculated by the following formula: recombination efficiency (%) = (CFUs with Cam/CFUs without Cam) × 100.

### Genetic Manipulations

*Escherichia coli* plasmids were isolated by the alkaline lysis method, and the chromosomal DNA of *E. coli* or *M. xanthus* was extracted using a bacterial genome DNA extraction kit (Tiangen, Beijing, China). Cloning of the genes *recA1*, *recA2*, and *lexA* from *M. xanthus* was performed according to the general steps (Ausubel et al., 1995). The genes were amplified by PCR and ligated into the pET15b expression plasmid. The primers used here are listed in Supplementary Table S2.

Mutant construction was performed using the markerless mutation in *M. xanthus* DK1622, with the pBJ113 plasmid using the Kan-resistant cassette for the first round of screening and the *galK* gene for the negative screening (Ueki et al., 1996). Briefly, the up- and downstream homologous arms were amplified with primers (listed in Supplementary Table S2) and ligated at the *Bam*HI site. The ligated fragment was inserted into the *Eco*RI/*Hind*III site of pBJ113. The resulting plasmid was introduced into *M. xanthus* via electroporation (1.25 kV, 300 W, 50 mF, and 1 mm cuvette gap). The second round of screening was performed on CYE plates containing 1% galactose (Sigma). The *recA1* (named RA1) and *recA2* (named RA2) mutants were identified and verified by PCR amplification and sequencing.

We attempted to construct the *recA1/recA2* double mutant from the single deletion mutant (RA1 or RA2) using the same procedure as described above, but all failed.

### RNA Extraction, RT-PCR, and RNA-Seq Assay

Total RNA of *M. xanthus* cells was extracted using RNAiso Plus reagent following the manufacturer’s protocol (Takara, Beijing, China). cDNA synthesis was performed using the PrimeScript RT Reagent Kit with random primers. The synthesized cDNA samples were diluted five times prior to RT-PCR. The primers were designed for *lexA*, *recA*1, and *recA*2 (Supplementary Table S2). RT-PCR was accomplished using the SYBR Premix Ex Taq Kit (Takara, China) on an ABI StepOnePlus Real-Time PCR System (ThermoFisher Scientific, United States). Gene expression was normalized to the *gapA* expression and calculated using the equation: change (*x*-fold) = 2^–ΔΔCt^ (Schefe et al., 2006).

RNA sequencing was conducted by Vazyme (Beijing, China). Three independent repeats are set for each sample. All the up- and downregulated genes were obtained by comparing the expression of the genes with that of the control, and their gene functions were annotated using the NR, GO, and KEGG databases.

### Protein Expression, Purification, and Characterization

The constructed expression plasmids with *recA1*, *recA2*, or *lexA* were introduced into *E. coli* BL21(DE3) competent cells. Protein expression was induced with 1 mM IPTG and purified with Ni-NTA agarose according to the manual of the Ni-NTA purification system (Invitrogen). After overnight dialysis with storage buffer [20 mM Tris–HCl (pH 7.2), 150 mM NaCl, 0.1 mM DTT, 0.1 mM EDTA, and 50% glycerol], the purified proteins were quantified and stored at −80°C.

The ATPase activity of RecA protein was determined in the presence or absence of DNA according to the methods described previously (Sheng et al., 2005). The final reaction mixture in a 2-ml volume contained: 20 mM Tris–HCl (pH 7.4), 10 mM NaCl, 5 mM MgCl_2_, 2 mM KCl, 3 mM ATP (Sigma), 1 mM CaCl_2_, 1 mM DTT, and 2% glycerol. The mixture was preheated to 32°C before the addition of RecA and DNA. ATPase activity was determined by measuring the free phosphate ion (Pi) released from ATP using an ultramicro ATPase activity detection kit (Nanjing Jiancheng Bioengineering, Nanjing, China).

*In vitro* LexA cleavage analysis was performed as described previously (Sheng et al., 2005).

D-loop assays for strand assimilation were performed according to the previously described methods (Cloud et al., 2012; Huang et al., 2017) with some modifications. Briefly, 0.2 μM RecA and 10 nM ^32^P-labelled ssDNA was combined in 9 μl of reaction buffer containing 25 mM Tris–HCl (pH 7.5), 75 mM NaCl, 5 mM MgCl_2_, 3 mM ATP, 1 mM DTT, and 1 mM CaCl_2_ and incubated at 37°C for 5 min. Then, 1 μl of RF M13 plasmid was added to a final concentration of 1 μM, and the incubation at 37°C was continued for 20 min. The reaction was stopped by adding sodium dodecyl sulfate to 0.5% and proteinase K to 1 mg/ml. The deproteinated reaction products were run on a 0.9% agarose 1× TAE gel and visualized using autoradiography with phosphor screen.

*In vitro* DNA strand-exchange reactions were performed as described previously (Sheng et al., 2005).

## Results

### Duplicate *recA* Genes in *M. xanthus* Are Both Induced by UV Irradiation

**FIGURE 1:**
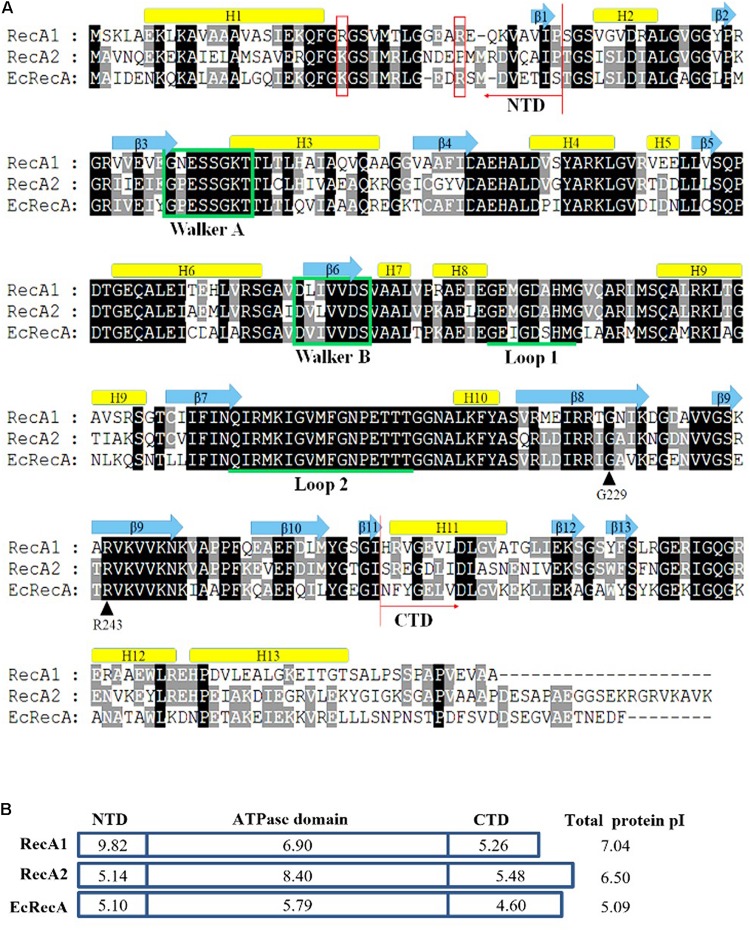
Amino acid sequence comparison of RecA proteins. **(A)** Alignment of *M. xanthus* RecA1 and RecA2 and *E. coli* RecA (EcRecA, b2699). Positions of the N-terminus (NTD) and the C-terminus (CTD) domains are indicated with red arrows, respectively. Their secondary structures all contain 13 alpha-helixes and 13 beta-sheets, which are indicated above their corresponding amino acid sequences. The ATP binding Walker A and B motifs are marked in green frames, and the putative DNA binding sites Loop L1 and L2 are indicated by underlines of the corresponding amino acid sequences. Two reported LexA binding sites (G229 and R243) are indicated by black arrows. K23 and R33 in the N-terminal region of EcRecA are labeled with red boxes. **(B)** The pI features of the domains of the three RecA proteins. The theoretical pI values were computed using ExPASy online tools (Compute pI/Mw).

The two RecA proteins of *M. xanthus* DK1622 are highly conserved and are both homologous to the RecA protein of *E. coli* K12 (EcRecA). The two RecA coding genes have high G+C contents (66 and 65%, respectively); the amino acid identity of RecA1 and RecA2 is 64.6%, and they are 59.36 and 62.04% to EcRecA, respectively. Similar to EcRecA (Story et al., 1992; Lee and Wang, 2009), RecA1 and RecA2 consist of three structural domains, a small N-terminal domain (NTD), a core ATPase domain (CAD), and a large C-terminal domain (CTD). CAD contains the conserved ATPase Walker A and Walker B domains and L1 and L2 DNA-binding domains (Figure 1A). The CAD of RecA1 and RecA2 are highly conserved, while the NTD and CTD are varied. Compared with EcRecA, the two RecA proteins of *M. xanthus* have more basic amino acids, and the theoretical isoelectric points [pI, calculated by online software (ExPASy – Compute pI/Mw tool)] of RecA1 and RecA2 are 7.04 and 6.5, respectively; EcRecA is more acidic, with a theoretical pI of 5.09 (Figure 1B). Differences in the amino acid composition suggested that the RecA1 and RecA2 proteins might vary in their functions.

**FIGURE 2:**
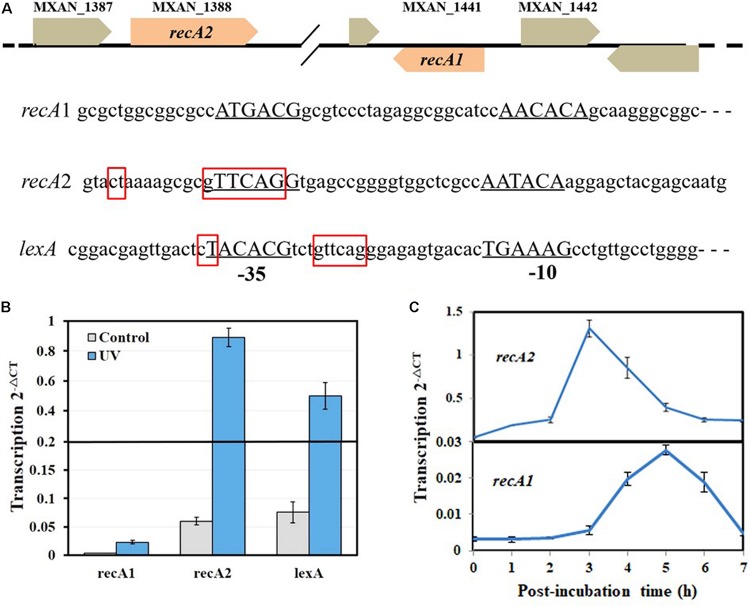
Organization and UV inducibility of the *recA1* and *recA2* genes of *M*. *xanthus* DK1622. **(A)** Schematic gene location and promoter alignment of *M*. *xanthus recA1* and *recA2*. RNA polymerase binding sites (–10 and –35 regions) are underlined, and the corresponding nucleotide sequences are in capitals. The SOS box regions are framed in red squares, and the sequence in the promoter of the *lexA* gene (*MXAN_4446*) was used as a control. **(B)** UV inducibility of *recA1* and *recA2*. The strains were incubated for 4 h after UV irradiation treatment with a dose of 15 J/m^2^ in a UV crosslinking machine and used to detect transcription of *recA1* and *recA2* by RT-PCR. *lexA* was used as a control. **(C)** The induction time points of *recA1* and *recA2*. After being exposed to UV irradiation at a dose of 15 J/m^2^, the cell cultures were post-incubated at 30°C, sampled at certain intervals to extract the total RNA for RT-PCR. The error bars in panels **B** and **C** represent means ± SEM (*n* = 3, *p* < 0.05 versus inner reference).

The SOS response of *M. xanthus* cells to DNA damage can be divided into LexA-dependent and -independent types (Campoy et al., 2003). The LexA-dependent SOS genes, e.g., *lexA*, typically possess a LexA-box sequence in their promoters. Each of the two *recA* genes of *M. xanthus* has its own promoter and is not a part of an operon. A typical LexA-box sequence was found in the promoter of *recA2* but not in the *recA1* promoter (Figure 2A). Previous studies reported that *recA2* was obviously induced by nalidixic acid and mitomycin C but that *recA1* was not induced by mitomycin C (Norioka et al., 1995; Campoy et al., 2003). We treated *M. xanthus* cells with 15 J/m^2^ UV irradiation, which is also a normal induction agent for investigating the bacterial SOS response (Courcelle et al., 2001; Rastogi et al., 2010; Richa et al., 2015). RT-PCR revealed that *lexA* and *recA2* were upregulated by 8.3 times and 10.7 times, respectively, 4 h after UV irradiation at 15 J/m^2^ (Figure 2B). Interestingly, the *recA1* gene was also UV-induced by 6.4 times. The basal expression level of *recA1* was very low and was less than one-tenth that of *recA2*. The low expression level of *recA1* might be the reason why the expression of *recA1* was not detected by Northern blotting (Norioka et al., 1995). The generation time of *M. xanthus* cells is about 3–4 h in the exponential growth stage. We found that the induction of *recA2* peaked at approximately 3 h after UV treatment, whereas the induction time of *recA1* was delayed and peaked 5 h after the treatment (Figure 2C). The different expression levels and induction time points implied that the two RecA proteins participate in the repair of different types of DNA damage caused by UV irradiation.

### Inactivation of *recA2* Compromises the Growth of *M. xanthus* Cells

**FIGURE 3:**
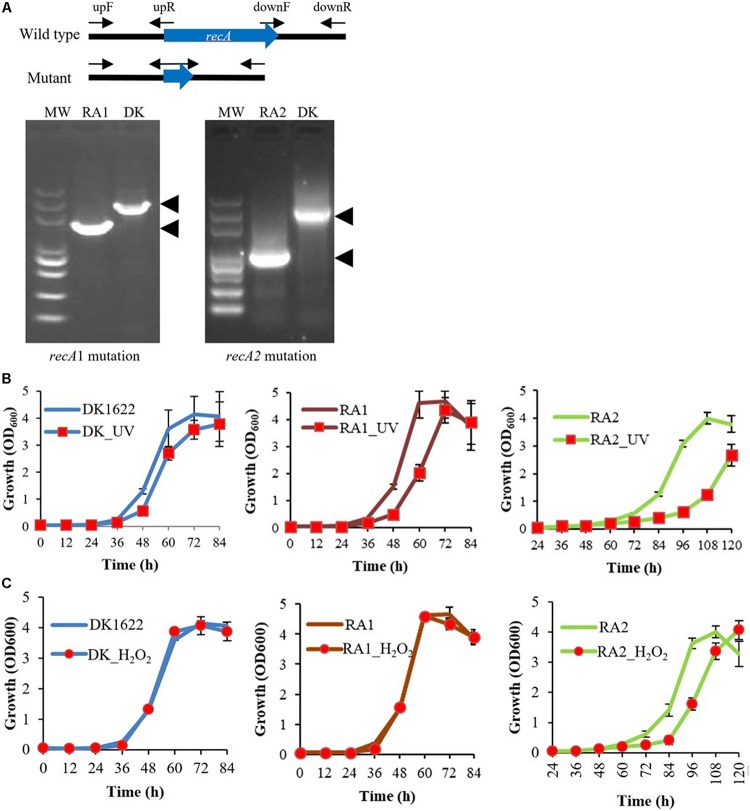
Mutations of *recA1* and *recA2*, and their effects on the growth of *M. xanthus*. **(A)** Deletion of *recA1* or *recA2* in *M. xanthus* DK1622, using the markerless knockout plasmid pBJ113, producing the RA1 or RA2 mutants. The deletion was verified by PCR using their primer pairs (upF/downR) and sequencing. **(B)** Separate growth comparisons of DK1622, RA1, and RA2 with and without UV treatment at a dose of 15 J/m^2^. **(C)** Separate growth comparisons of DK1622, RA1, and RA2 with and without the H_2_O_2_ treatment at a final concentration of 3 mM for 15 min. The error bars indicate the SEM for six replicates.

In previous studies, *recA2* deletion mutants were not obtained in either *M. xanthus* or *B. megaterium* (Norioka et al., 1995; Campoy et al., 2003; Nahrstedt et al., 2005). However, in this study, we successfully obtained the deletion mutant of both *recA1* and *recA2* in *M. xanthus*, named RA1 and RA2, respectively (Figure 3A). According to the two-step screening method employed, the acquisition probability from reverse screening was ∼10^–6^ for the deletion of *recA1* and ∼3.3 × 10^–10^ for the deletion of *recA2*, and this may be the reason why it is difficult to make a *recA2* mutant. At present, there is no evidence of a suppressor mutation, which may be required to achieve the deletion of *recA2*. However, although more evidence is needed, we speculate that the difficulty in the screening of RA2 is probably related to the function of RecA2 in growth. We also tried to construct the double knockout mutant of *recA1* and *recA2* but failed, and this might be because the double mutation had a serious impact on cell survival and was synthetically lethal. *recA1* deletion had no significant effects on cellular growth, but deletion of *recA2* caused the mutant to have a long lag phase. After the lag phase, growth of the RA2 mutant did not slow down significantly in the logarithmic phase, and the mutant culture reached a similar density as wild-type DK1622 (Figures 3B,C).

When treated with 15 J/m^2^ UV irradiation, compared with those without UV treatment, the growth abilities were delayed in DK1622, RA1, and RA2 cells, and the growth delay was more notable in RA2 (Figure 3B). When treated with 3 mM H_2_O_2_ for 15 min, DK1622 and RA1 cells showed almost the same growth curve, while the growth of RA2 cells was delayed significantly compared with that of the strains without the treatment (Figure 3C). The results demonstrated that *recA2*, but not *recA1*, is an important factor for cell growth after UV irradiation and oxidation damage.

### *recA1* and *recA2* Are Separately Crucial for UV Resistance and H_2_O_2_ Resistance

**FIGURE 4:**
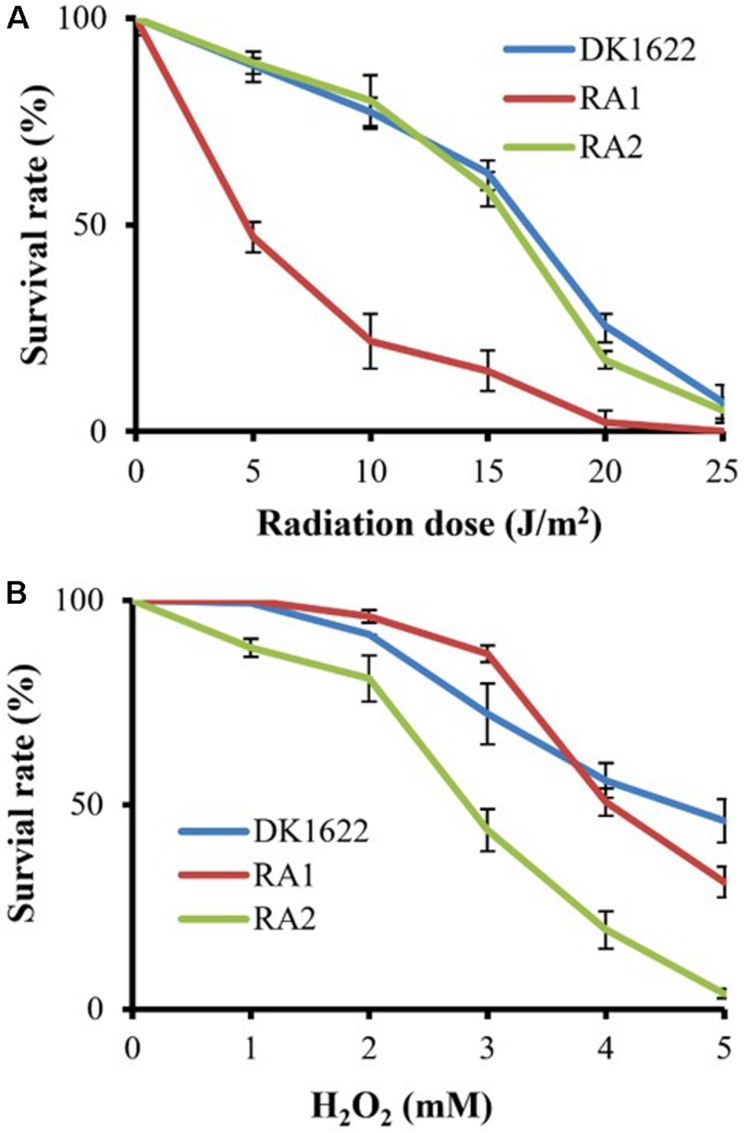
Survival of *M. xanthus* wild-type strain DK1622 and the mutants RA1 and RA2. **(A)** Survival curves after exposure to UV irradiation at different dosages (0–25 J/m^2^). **(B)** Survival curves after hydrogen peroxide treatment at different concentrations (0–5 mM). The percentage of surviving cells was calculated by comparing with the corresponding non-treated cells. The error bars indicate the SEM for six replicates.

We measured the survival rates of the wild-type strain and the *recA* deletion mutants treated with different dosages of UV irradiation (0–25 J/m^2^) and H_2_O_2_ (1–5 mM). All three strains had decreased survival rates with increasing UV irradiation or H_2_O_2_ concentration. Interestingly, the survival rate of RA1 cells decreased more significantly than that of RA2 at each UV-radiation dosage, which had a highly similar survival curve to the wild-type strain (Figure 4A). In addition, the survival rate of RA2 cells decreased more significantly at each H_2_O_2_ concentration than that of RA1 and DK1622 cells, which showed similar survival curves when treated with hydrogen peroxide (Figure 4B). Thus, RecA1 is needed for the survival of *M. xanthus* cells under UV irradiation, which is similar to that of EcRecA (Alexseyev et al., 1996), whereas RecA2 is involved in tolerance to H_2_O_2_ damage in cells.

### RecA1, Not RecA2, Is Responsible for HR and LexA-Dependent SOS Induction

**FIGURE 5:**
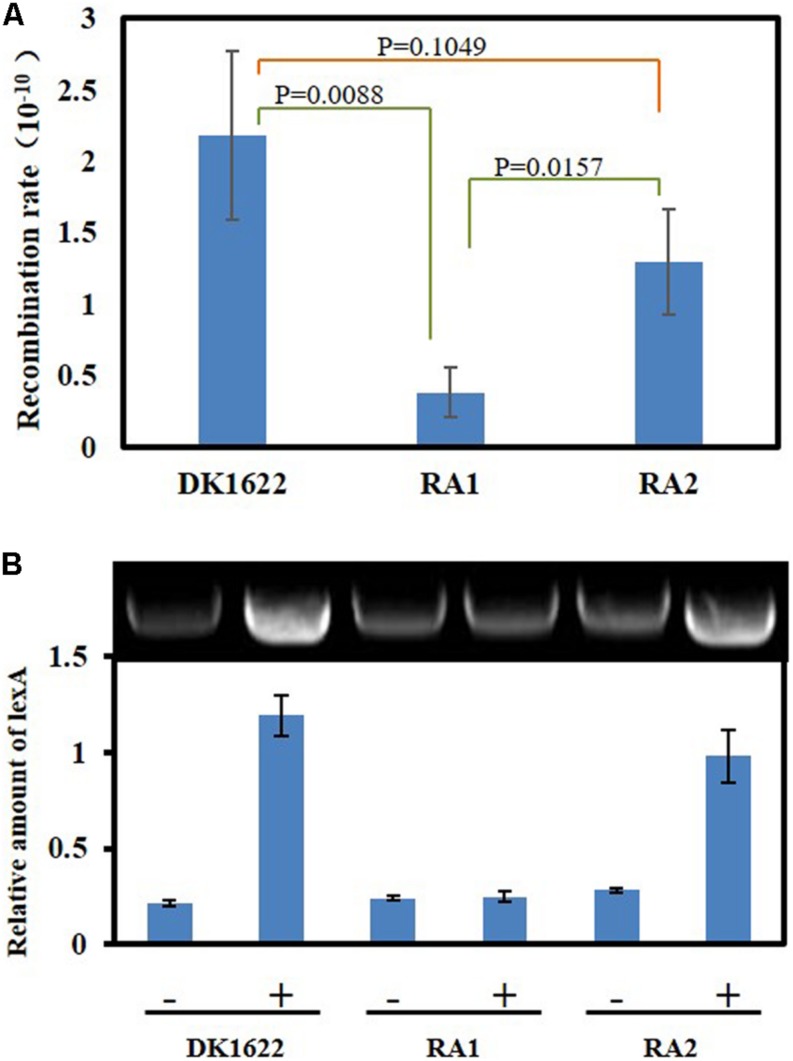
Intracellular DNA recombination rate and induction analysis of the *lexA* gene. **(A)** The cellular recombination rate of DK1622, RA1, and RA2. The two ends of the resistance gene (*kan*) have homologous DNA sequences with the two ends of the insertion site, respectively, and the *kan* gene is transferred into the *M. xanthus* through electrical transformation. The recombination rate was calculated by measuring the proportion of kanamycin-resistant colonies. **(B)** The inducibility of the *lexA* gene. *Myxococcus lexA* is a known SOS gene induced through LexA-dependent SOS response, and herein, its UV inducibility represents the activation of LexA-dependent SOS response. The strains were irradiated with 15 J/m^2^ UV irradiation (+) or mock treatment (–), and the transcription of *lexA* was determined by RT-PCR.

DNA HR and SOS induction are the two main cellular functions of the RecA proteins (Cox, 2003). We analyzed the *in vivo* integration abilities of an antibiotic resistance gene into the genomes of the DK1622, RA1, and RA2 strains. Calculated from the appearance of resistant colonies, the recombination rate of RA1 cells was significantly lower than that in either DK1622 (*p* = 0.0088) or RA2 (*p* = 0.0157) cells, while the differences between the recombination rates of RA2 and DK1622 cells were not significant (*p* = 0.1049) (Figure 5A). The results showed that *recA1* is important for the recombination process in *M. xanthus*.

Previous studies indicated that the expression of *lexA* is induced by the LexA-dependent SOS response in *M. xanthus* (Norioka et al., 1995). We compared the transcription of *lexA* in the *M. xanthus* DK1622, RA1, and RA2 strains in response to the 15 J/m^2^ UV irradiation treatment. The RT-PCR results showed that *lexA* could be induced by UV in both DK1622 and RA2 but not in the RA1 mutant (Figure 5B). Thus, the deletion of *recA1*, rather than *recA2*, affected the UV-induction of *lexA*, i.e., RecA1 is responsible for LexA-dependent SOS induction.

### RecA1 and RecA2 Both Have ss- and ds-DNA Promoted ATPase Activities

**FIGURE 6:**
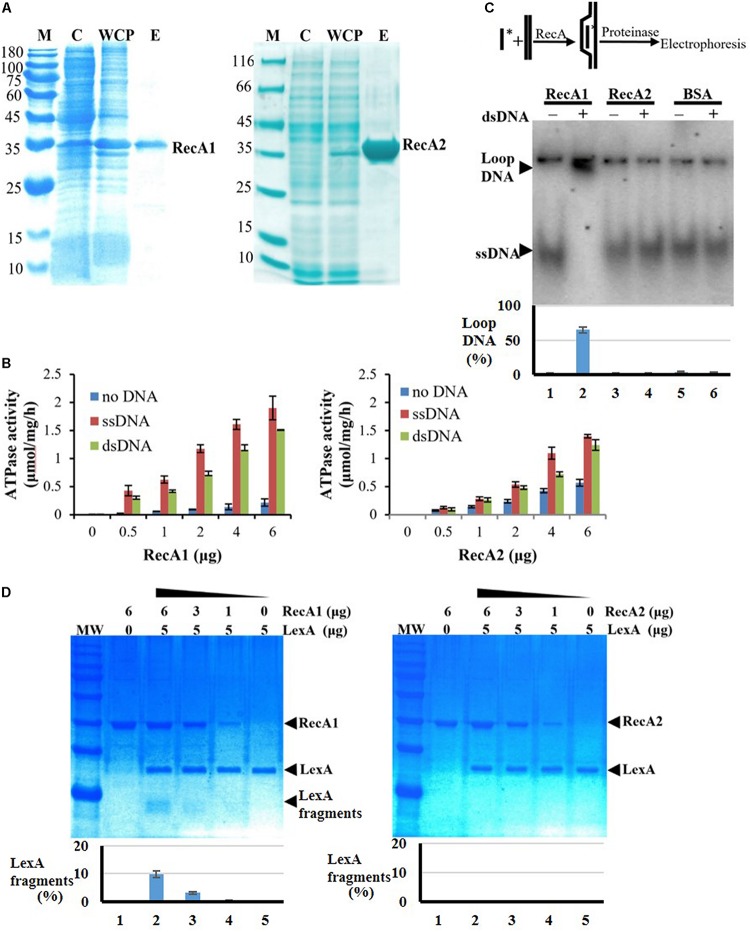
Expression and activity analysis of RecA proteins. **(A)** Expression and purification of RecA1 and RecA2. M, marker; C, control; WCP, whole-cell protein; E, eluent of purified protein. **(B)** Assays of ATPase activities. The ATPase activity was determined by measuring free phosphate ion (Pi) released from enzymolysis of ATP. The error bar is calculated from three independent repeats. **(C)** D-loop assay. A 60-nt ^32^P-labeled ssDNA fragment and a superhelical dsDNA (RF M13) sequence were mixed and incubated with and without the addition of purified RecA1 or RecA2 proteins. If the protein has HR activity, the homologous pairing reaction will be initiated, thus forming the ssDNA–dsDNA complex. Bovine serum albumin (BSA) was used as a control. Relative DNA-labeled intensities of the bands were quantified by a Gel-Doc image analysis system (Bio-Rad). The percentage of loop-DNA-labeled intensity in the total labeled intensity (including the labeled strength in ssDNA, loop-DNA, and residual DNA in the origin) was used to quantitate the RecA activity. **(D)** The promotion ability of RecA1 (left) or RecA2 (right) on the cleavage of LexA proteins. The MxLexA protein was incubated with gradient concentrations of RecA1 or RecA2 proteins in the presence of ssDNA and ATP. Reactions were stopped and visualized on a 1.2% SDS-PAGE gel stained with Coomassie brilliant blue. The bands were quantified by computerized image analysis (Bio-Rad), and the percentage of LexA fragments in the total LexA signal in every lane was used to quantify the ability of RecA to stimulate LexA autocleavage.

We further expressed and purified the RecA1 and RecA2 proteins (Figure 6A) and measured their *in vitro* ATPase activities by the quantitative analysis of inorganic phosphorus released from ATP hydrolysis (Figure 6B). In the reaction mixture without the addition of DNA, RecA1 and RecA2 both exhibited low ATPase activities, and the ATPase activity of RecA2 was somewhat higher than that of RecA1. For example, a microgram of purified RecA2 released 0.1428 nanomole Pi in an hour, which is approximately 2.44 times the hydrolysis capacity of RecA1 on ATP (0.0586 nmol Pi/μg/h). The addition of DNA, especially ssDNA, markedly promoted the ATPase activity of both RecA1 and RecA2, and this is consistent with the functionality of classic RecA proteins (Cox, 2003; Greene, 2016). Thus, RecA1 and RecA2 are both DNA-dependent (more dependent on ssDNA) ATPase enzymes. In the presence of DNA (dsDNA or ssDNA), the increase in the ATPase activity of RecA1 was higher than that of RecA2. For example, the ATPase activity of 1 ng RecA1 increased by 10.69 times (from 0.0586 to 0.6265 nmol Pi/μg/h) with the addition of ssDNA, while the increase of that in RecA2 was only double (from 0.1428 to 0.2857 nmol Pi/μg/h). Similarly, the addition of dsDNA increased the ATPase activities of RecA1 and RecA2 by 6.89 times (from 0.0586 to 0.4038 nmol Pi/μg/h) and 1.86 times (from 0.1428 to 0.2658 nmol Pi/μg/h), respectively.

### RecA1, but Not RecA2, Has *in vitro* HR Capacity and Activates LexA Autolysis

Strand invasion or D-loop formation is a central step in HR and is one of the most common biochemical assays for characterizing the activity of RecA-type recombinase (Cox, 2003; Greene, 2016; Huang et al., 2017). We analyzed the *in vitro* recombination activities of RecA1 and RecA2 in a DNA strand recombination reaction system containing ^32^P-ssDNA and homologous plasmid dsDNA. The reaction products were separated by agarose gel electrophoresis, and a lagged radiolabeling band appeared in the lane containing purified RecA1 but that with not RecA2 (Figure 6C). Furthermore, DNA strand exchange (DSE), another characteristic reaction for RecA-catalyzed HR with single-stranded circular DNA and its homologous double-stranded linear DNA, was used to identify the recombination activity of RecA1 and RecA2 (Supplementary Figure S5). The joined molecule DNA (jmDNA) appeared and increased as a recombinant product with the gradient addition of the RecA1 protein but not with that of the RecA2 protein. The above results indicated that RecA1, but not RecA2, has HR activity in *M. xanthus*, and these results are consistent with the *in vivo* recombination results (Figure 5A).

RecA promotes LexA autolysis at a specific site, thereby enabling the expression of SOS genes inhibited by LexA (Janion, 2008; Kovačič et al., 2013). We monitored the LexA cleavage activity promoted by RecA1 and RecA2, using the *M. xanthus* LexA protein as a substrate. The results showed that the LexA autolysis fragments were detected in the reaction with RecA1 but not that with RecA2 (Figure 6D). Thus, RecA1 participated in the LexA-dependent SOS induction reaction, and this is also consistent with the RA1 mutant losing the induction ability of the SOS gene *lexA* (Figure 5B).

## Discussion

RecA is an ATP-dependent recombinase central to DNA HR and activation of the LexA-dependent SOS response. Although the *recA* gene is duplicated in some bacterial cells, its functions have not been investigated. In *M. xanthus* DK1622, the expression of *recA1* is very low and is less than one-tenth of that of *recA2*. The two *recA* genes are both inducible by UV irradiation, but the induction of *recA2* was significantly earlier than that of *recA1* and recN (Supplementary Figure S6). Generally, the DNA repair genes expressed in the early and late stages of SOS are responsible for the error−free repair and maintenance processes and error-prone DNA synthesis against serious DNA damage, respectively (Kuzminov, 1999; Janion, 2008; Maslowska et al., 2019). Thus, the two RecA proteins are both involved in UV resistance, probably for different lesions caused by UV irradiation (Sinha and Häder, 2002); RecA2 is involved in the early repair processes, and RecA1 is involved in serious DNA-damage repair, i.e., post-replication repair. The deletion of *recA2* caused the mutant to have a long lag phase, but the *recA1* deletion had no significant effect on cellular growth. It is known that the growth lag phase is an adaptation period of bacterial cells to changes in temperature and nutrients in new environments (Monod, 1949; Vermeersch et al., 2019), macromolecule damage repair, and protein misfolding accumulated during cell arrest (Dukan and Nyström, 1998; Saint-Ruf et al., 2007; Rolfe et al., 2012; Bertrand, 2019), and enzyme preparation for rapid growth in the logarithmic phase (Monod, 1949; Rolfe et al., 2012). Thus, RecA2, instead of RecA1, plays a crucial role in the repair process required for cellular growth. Similar to the classic bacterial RecA, RecA1 possesses DNA recombination activity and SOS gene induction ability, which are required for survival under UV irradiation. However, RecA2 has lost its HR and SOS gene induction abilities but has evolved to play roles in the regulation of gene expression for cellular growth as well as cellular survival under oxidation pressure by hydrogen peroxide. This is the first study to clearly determine the divergent functions of duplicated *recA* genes in bacterial cells.

**FIGURE 7:**
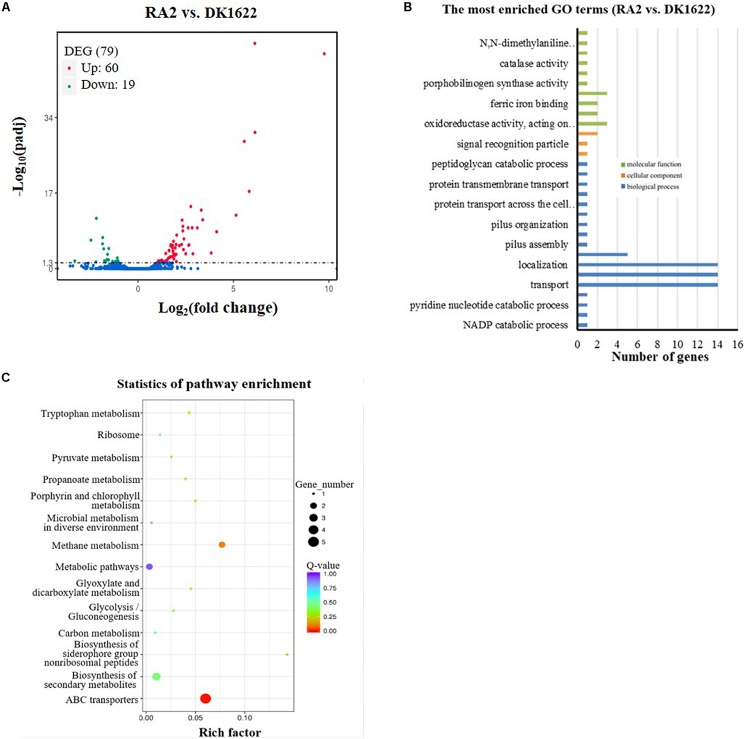
Comparison of transcriptomes between the *recA2* mutant (RA2) and the wild-type strain (DK1622). **(A)** Volcano plot of differentially expressed gene (DEG) distributions. Red dots and green dots represent the up- and down-regulated genes with significant differences, respectively (*p*_adj_. < 0.05). The blue dots represent the genes that have not changed significantly. **(B)** The distribution of GO category and **(C)** pathways of DGEs between RA2 and DK1622. Enriched GO is shown in three categories: biological process (blue), molecular function (green), and cellular component (orange).

To obtain more clues about the potential mechanisms of RecA2 in *M. xanthus*, we compared the transcriptomes of the *recA2* mutant strain (RA2) and wild-type strain DK1622. Overall, 79 genes were found to be differentially expressed (*p*_adj_. < 0.05) by the deletion of *recA2*, and this included 60 upregulated genes and 19 downregulated genes (Figure 7A; for details, refer to Supplementary Table S3). Gene ontology (GO) enrichment analysis based on the KEGG database showed that the differentially expressed genes (DEGs) were assigned to 30 GO terms in the categories of biological process, cellular component, and molecular function (Figure 7B). Obviously, the biological process DEGs formed the largest group and included 17 GO terms, followed by the molecular function (10 GO terms) and cellular component (three GO terms) groups (Figure 7B). The DEGs were mainly enriched in two functional regions. One is related to transport and location, including the categories of transport (14 genes), localization and establishment of localization (28 genes), transmembrane transport (five genes), and protein transmembrane transport (three genes). The other category is related to antioxidation and includes the categories of oxidoreductase activity (three genes), peroxiredoxin activity (two genes), ferric iron binding (two genes), antioxidant capacity (three genes), and catalase (one gene). These DEGs were significantly enriched in ABC transporters and several metabolism-related pathways, such as methane metabolism, biosynthesis of secondary metabolites, and metabolic pathways (Figure 7C). Combining this with the experimental results presented in this study, we propose that the function of *recA2* is mainly related to cellular transportation and antioxidation, which are required for the normal growth of cells.

RecA1 and RecA2 are both homologous proteins with *E. coli* RecA. They both retain the DNA-dependent ATPase activity and self-aggregation ability of *E. coli* RecA (Figure 6B and Supplementary Figure S7), and the interaction between RecA1 and RecA2 may even be due to the conserved self-aggregation sites. On the other hand, these two proteins also showed significantly different activities in DNA recombination and LexA autocleavage. Amino acid sequence alignment showed that the RecA1 and RecA2 amino acid sequences are highly similar in the CAD, and are mainly varied in the NTD and CTD (Figure 1A). Lys23 and Arg33 in the N-terminal region are both necessary for the nucleoprotein filament of RecA–ssDNA to capture homologous dsDNA (Lee and Wang, 2009). The corresponding amino acids at the two sites are both alkaline arginine residues in RecA1, which is consistent with that in EcRecA. In RecA2, however, the amino acids at the two sites are arginine and proline, respectively (Figure 1A). We aligned the N-terminal sequences of 11 reported bacterial RecA proteins. The amino acids at the corresponding 23rd site are all alkaline amino acids but are less conserved at the 33rd site (Supplementary Figure S1). Nine RecAs, including RecA1 of *M. xanthus*, have positively charged residues (Arg or Lys) at the 33rd site, while three RecAs, including RecA2 from *M. xanthus*, RecA from *Prevotella ruminicola* (Aminov et al., 1996), and RecA from *Borrelia burgdorferi* (Huang et al., 2017), did not have the positively charged residues at this site. RecAs with an alkaline amino acid at the 33rd site all have DNA recombination activity (Sano and Kageyama, 1987; Nussbaumer and Wohlleben, 1994; Umelo et al., 1996; Cox, 1999; Kim et al., 2002; Orillard et al., 2011; Grove et al., 2012; Carrasco et al., 2019). However, similar to RecA2, RecAs from *P. ruminicola* and *B. burgdorferi* were reported to have no anti-ultraviolet radiation ability (Aminov et al., 1996; Huang et al., 2017). The results presented in this study demonstrate that RecA2 of *M. xanthus* evolved to affect the genes for cellular transportation and antioxidation, which are obviously related to damage repair for cellular growth.

As in *E. coli* RecA (Lusetti et al., 2003; Kovačič et al., 2013), RecA 1 and RecA2 both have conserved LexA binding sites, including Gly229 and Arg243, in their C-terminal regions and 10 neighboring amino acids (Figure 1A), which, however, does not explain the differences between the two proteins in promoting LexA autolysis. Notably, while the three domains of EcRecA are all acidic, the NTD of RecA1 and the CAD of RecA2 are alkaline, with pI values of 9.82 and 8.40, respectively. Accordingly, RecA1 forms more negative charges on the outer side of the polymer, while RecA2 forms more negative charges on the inner side of the helical structure (Supplementary Figure S2). We noted that, unlike the *E. coli* LexA (EcLexA) protein, which is an acidic protein [theoretical pI is 6.23, calculated by online software (ExPASy Compute pI/Mw tool)], *M. xanthus* LexA (MxLexA) is a basic protein, and its theoretical pI is 8.77. EcLexA and MxLexA are conserved in their catalytic and DNA binding domains, and the differences between the two proteins lie mainly in the linker region (Supplementary Figure S3). The EcLexA linker contains more acidic amino acids (theoretical pI = 3.58), while the linker of MxLexA contains more basic amino acids (theoretical pI = 8.75). In addition, MxLexA has two more fragments flanking the linker sequence. The additional fragment at the N-terminal side destroys the β2 folding structure and further lengthens the irregular linker of MxLexA, leading to a long irregular chain containing more basic amino acids (theoretical pI = 12.01). According to the binding mode between EcLexA and EcRecA (Kovačič et al., 2013), the linker region of LexA is close to the inner groove of the RecA protein filament (Supplementary Figure S4). The inner helix part of RecA2 (in the CAD) is alkaline (Supplementary Figure S2B), which hinders MxLexA binding to the RecA filament and thus hinders its ability to promote MxLexA self-cleavage.

Myxobacteria has a relatively large genome size (9–14 Mbp) and contains many DNA repeats (Goldman et al., 2006; Andersson and Hughes, 2009; Han et al., 2013). These repetitive DNA fragments are potential substrates for RecA-catalyzed HR. Functional divergence of duplicate RecA proteins and low expression of the recombination enzyme RecA1 reduce the DNA recombinant activity without affecting other cellular repair functions in *M. xanthus* (such as the functions carried out by RecA2). In the sequenced myxobacterial genomes (Supplementary Table S4), all the strains, except *Anaeromyxobacter*, harbor two *recA* genes, and their amino acid sequences are highly conserved. For example, the amino acid identities of RecA1 and RecA2 of all *Myxococcus* are >89.4 and 93.6%, respectively. The *Anaeromyxobacter* strains have a single *recA* gene in their genomes; however, their genomes are small (5.0–5.2 Mbp) and possess few repetitive sequences. We propose that the functional divergence and expression regulation of duplicate RecA proteins might be a strategy for bacteria with a large number of repetitive sequences in their large genomes to avoid incorrect recombination.

## Data Availability Statement

All datasets generated for this study are included in the article/Supplementary Material.

## Author Contributions

D-HS and Y-ZL designed the experiments. D-HS, Y-XW, MQ, and J-YZ performed the experiments. D-HS, X-JY, and Y-ZL analyzed the data. D-HS and Y-ZL wrote the manuscript.

## Conflict of Interest

The authors declare that the research was conducted in the absence of any commercial or financial relationships that could be construed as a potential conflict of interest.
