# Supplementary material for: Functional Division Between the RecA1 and RecA2 Proteins in Myxococcus xanthus
Source: Front Microbiol. 2020 Feb 12;11:140. doi: 10.3389/fmicb.2020.00140 (PMC7029660; doi:10.3389/fmicb.2020.00140)
Supplement: Supplementary file 1 [file Data_Sheet_1.pdf]

Table S1. Strains and plasmids used in this study

Strains or plasmids	Genotype or description	Source or references
**Strains**		
M. xanthus		
DK1622	Wild-type strains	D. Kaiser, University of Stanford
RA1	DK1622 (ΔrecA1)	This study
RA2	DK1622 (ΔrecA2)	This study
E. coli		
DH5α	F^-80dlacZΔM15 Δ(lacZYA$^-$argF)U169 deoR recA1 endA1 hsdR17 ($r_k^- m_k^+$) phoA supE44 λ^-thi-1 gyrA96 relA1	Takara
JM109	recA1, endA1, gyrA96, thi-1, hsdR17, supE44, relA1, Δ(lac$^-$proAB)/F'[traD36, proAB$^+$, lacIq, lacZΔM15]	Promega
BL21(DE3)	F^-lon ompT hsdSB (rB-, mB-) dcm gal dcm(DE3)	This study
BL2/pET15recA1	Expression strain of recA1 with plasmid pET15recA1	This study
BL21/pET15recA2	Expression strain of recA2 with plasmid pET15recA2	This study
**Plasmids**		
pBJ113	Gene replacement vector with KG cassette, Kan^r	Z.M. Yang, Virginia Tech
pBJ-recA1	Upstream and downstream homologous arms of recA1 inserted into EcoRI/HindIII site of pBJ113, Kan^r	This study
pBJ-recA2	Upstream and downstream homologous arms of recA2 inserted into EcoRI/HindIII site of pBJ113, Kan^r	Wu & Kaiser, 1995
pET15recA1	Recombination plasmid with a recA1 gene inserted into NdeI/BamHI sites of pET15b, Amp^r	This study
pET15recA2	Recombination plasmid with a recA1 gene inserted into NdeI/BamHI sites of pET15b, Amp^r	This study
pET15lexA	Recombination plasmid with a lexA gene inserted into NdeI/BamHI sites of pET15b, Amp^r	This study
M13mp	a filamentous E. coli bacteriophage, used for DNA recombination assay	NEB

Table S2. Primers used in this study

Primer name	Primer sequence (5'-3')
**MXAN_1441_UF**	ATGGATCCGCCGCCGCCACTGCCTTCA
**MXAN_1441_UR**	GTATCCACACCCGTCACTTCC
**MXAN_1441_DF**	GACCGCACGGGGCTCTTCAAT
**MXAN_1441_DR**	ATGGATCCTAGACGGAGGACGCCAACAC
**MXAN_1388_UF**	GACTGGTGGATGCGAAGGGACG
**MXAN_1388_UR**	TAGGATCCATGATGGACCCCTTGCCGAACTG
**MXAN_1388_DF**	AAGGATCCGAAGGTGGCAGCGAGAAGCG
**MXAN_1388_DR**	GATGGTGAAGCGGTAGTAGTA
**Exp_1441_F**	TACATATGAGCAAGCTGGCGGAGAAG
**Exp_1441_R**	AAGGATCCCGGTCAAGCTGGACGTGTT
**Exp_1388_F**	CTCATATGGCCGTGAATCAGGAGAAGG
**Exp_1388_R**	TTGGATCCGGACTACTTCACGGCCTTCACAC
**Exp_4446_F**	TACATATGGAAGAGCTCACGGAACGCC
**Exp_4446_R**	AAGGATCCGGGACGGGTGGGGTGGACTA
**RTPCR_1441_F**	TCCAGGCGAGGCTGATGAGTC
**RTPCR_1441_R**	TCACCGTCCTTGATGTTGCCC
**RTPCR_1388_F**	CGTGAATCAGGAGAAGGAAAA
**RTPCR_1388_R**	TTCCCGAAGACCTCCACCACAC
**RTPCR_4446_F**	CTGTGCGGATGGCGTCTTCTTCA
**RTPCR_4446_R**	GGTGGAGATTCCCCTGCTGG

Table S3. List of differentially expressed genes between the transcriptomes of RA2 and DK1622.

Gene_id	Readcount of RA2	Readcount of DK1622	Log_2 Fold Change	Pval	Padj	Up or down	Gene annotation
MXAN_0009	878.87	371.6	1.24	1.02E-04	1.24E-02	up	MFS transporter
MXAN_0542	269.94	98.77	1.45	1.60E-04	1.83E-02	up	carbohydrate-binding protein
MXAN_1316	948.32	252.87	1.91	4.30E-06	6.41E-04	up	TonB-dependent receptor
MXAN_1318	1173.03	183.85	2.67	1.77E-08	4.80E-06	up	hemin-degrading factor
MXAN_1319	781.54	138.09	2.5	2.88E-06	4.67E-04	up	hemin ABC transporter substrate-binding protein
MXAN_1320	664.14	127.45	2.38	2.71E-07	5.35E-05	up	iron ABC transporter permease
MXAN_1321	557.35	124.05	2.17	2.84E-08	6.90E-06	up	hemin import ATP-binding protein HmuV
MXAN_1367	2730.65	753.6	1.86	2.15E-09	7.14E-07	up	prepilin-type cleavage/methylation domain-containing protein
MXAN_1369	1016.37	310.34	1.71	3.41E-08	7.78E-06	up	prepilin-type cleavage/methylation domain-containing protein
MXAN_1562	2340.05	343.94	2.77	8.55E-18	1.04E-14	up	DNA starvation/ stationary phase protection protein Dps
MXAN_1563	48548.43	5644.42	3.1	1.38E-12	6.74E-10	up	alkyl hydroperoxide reductase
MXAN_1564	77268.19	10809.55	2.84	1.17E-12	6.59E-10	up	peroxiredoxin
MXAN_1565	1065.7	481.25	1.15	1.65E-04	1.83E-02	up	ATPase AAA
MXAN_1709	226.26	56.72	2	3.76E-06	5.96E-04	up	DNA-directed RNA polymerase sigma-70 factor
MXAN_2217	2508.45	144.24	4.12	1.21E-11	5.18E-09	up	EamA/RhaT family transporter
MXAN_3461	564.4	229.34	1.3	1.98E-04	2.09E-02	up	KR domain-containing protein
MXAN_3462	2110.75	1059.45	0.99	5.13E-04	4.74E-02	up	KR domain-containing protein

MXAN_3640	2550.07	673.84	1.92	4.01E-06	6.10E-04	up	glutamate-1-semialdehyde aminotransferase
MXAN_3641	294.97	101.21	1.54	7.37E-05	9.31E-03	up	MFS transporter
MXAN_3914	907.34	284.12	1.68	1.71E-05	2.36E-03	up	PepSY domain-containing protein
MXAN_3915	2003.82	573.49	1.8	1.39E-05	1.99E-03	up	biopolymer transporter TonB
MXAN_4100	4757.48	2267.48	1.07	1.60E-04	1.83E-02	up	porphobilinogen synthase
MXAN_4290	768.06	328.8	1.22	1.40E-04	1.68E-02	up	thioesterase
MXAN_4291	1096	516.3	1.09	4.40E-04	4.12E-02	up	acyl carrier protein
MXAN_4389	69502.89	80.23	9.76	1.24E-52	4.53E-49	up	catalase
MXAN_4390	4558.82	65.17	6.13	9.28E-35	2.26E-31	up	ankyrin repeat domain-containing protein
MXAN_5244	811.59	56.95	3.83	1.69E-06	3.01E-04	up	DUF417 domain-containing protein
MXAN_5453	1842.88	545.53	1.76	3.40E-08	7.78E-06	up	MYXO-CTERM sorting domain-containing protein
MXAN_5454	1426.42	660.44	1.11	3.73E-04	3.58E-02	up	M36 family peptidase
MXAN_5856	7836.24	112.74	6.12	2.88E-55	2.10E-51	up	acetate--CoA ligase
MXAN_5857	205.92	3.63	5.82	2.87E-21	4.18E-18	up	DUF485 domain-containing protein
MXAN_5858	5441.09	114.83	5.57	1.29E-32	2.36E-29	up	cation/acetate symporter ActP
MXAN_5859	482.07	78.2	2.62	1.37E-12	6.74E-10	up	ion transporter
MXAN_6000	5281.69	1744.11	1.6	3.25E-05	4.32E-03	up	iron ABC transporter substrate-binding protein
MXAN_6805	1931.88	707.05	1.45	2.30E-06	3.99E-04	up	30S ribosomal protein S4
MXAN_6885	1304.96	326.91	2	1.34E-08	3.91E-06	up	DUF4105 domain-containing protein
MXAN_6911	2196.49	618.89	1.83	6.47E-07	1.18E-04	up	TonB-dependent receptor
MXAN_4914	1846.21	372.35	2.31	1.92E-14	1.28E-11	up	carbohydrate-binding protein
MXAN_1314	344.39	93.44	1.88	5.34E-05	6.96E-03	up	hypothetical protein
MXAN_1317	918.02	125.01	2.88	2.73E-06	4.64E-04	up	hypothetical protein

MXAN_1365	16250.01	4917.29	1.72	1.52E-08	4.27E-06	up	hypothetical protein
MXAN_1366	695.1	224.55	1.63	5.31E-07	9.93E-05	up	hypothetical protein
MXAN_1368	857.38	277.81	1.63	4.08E-07	7.85E-05	up	hypothetical protein
(1)	828.66	227.29	1.87	2.83E-06	4.67E-04	up	hypothetical protein
MXAN_1387	674.75	132.6	2.35	7.10E-12	3.24E-09	up	hypothetical protein
MXAN_1561	1190.1	233.49	2.35	5.25E-13	3.19E-10	up	hypothetical protein
MXAN_1689	3909.14	1116.51	1.81	9.86E-08	2.18E-05	up	hypothetical protein
MXAN_1697	277.44	85.51	1.7	1.66E-05	2.33E-03	up	hypothetical protein
MXAN_2219	230.2	60.82	1.92	7.76E-06	1.13E-03	up	hypothetical protein
MXAN_2812	13327.43	2740.24	2.28	1.22E-08	3.73E-06	up	hypothetical protein
MXAN_3191	4575.1	864.48	2.4	5.86E-10	2.14E-07	up	hypothetical protein
(2)	3376.62	322.61	3.39	1.45E-14	1.06E-11	up	hypothetical protein
MXAN_5266	1163.11	427.55	1.44	4.16E-04	3.95E-02	up	hypothetical protein
MXAN_5296	795.51	225.97	1.82	2.36E-08	5.93E-06	up	hypothetical protein
MXAN_5297	4936.81	497.04	3.31	6.63E-17	6.91E-14	up	hypothetical protein
MXAN_5300	3920.78	962.62	2.03	7.12E-11	2.89E-08	up	hypothetical protein
MXAN_5302	1163.92	301.47	1.95	1.05E-07	2.26E-05	up	hypothetical protein
MXAN_5855	1770.8	50.32	5.14	1.04E-15	9.46E-13	up	hypothetical protein
MXAN_7122	575.03	142.43	2.01	2.55E-04	2.55E-02	up	hypothetical protein
MXAN_6886	1818	289.55	2.65	2.12E-08	5.53E-06	up	hypothetical protein
MXAN_0133	1482.35	3063.96	-1.05	1.63E-04	1.83E-02	down	SGNH/GDSL hydrolase family protein
MXAN_0506	300.88	666.64	-1.15	2.48E-04	2.52E-02	down	NAD(P)/FAD-dependen t oxidoreductase
MXAN_2230	213.56	657.77	-1.62	7.40E-05	9.31E-03	down	LuxR family transcriptional regulator
MXAN_2249	57.46	194.32	-1.76	1.53E-04	1.80E-02	down	glycine betaine/L-proline ABC transporter ATP-binding protein
MXAN_2251	61.47	340.08	-2.47	1.12E-09	3.89E-07	down	glycine/betaine ABC transporter
MXAN_2399	674.06	1946.52	-1.53	1.47E-07	3.06E-05	down	serine/threonine protein kinase
MXAN_2840	278.67	967.24	-1.8	1.79E-07	3.63E-05	down	serine/threonine protein kinase
MXAN_3680	155.21	504.33	-1.7	3.58E-04	3.49E-02	down	gamma-glutamylcyclotr ansferase
MXAN_5560	544.97	5397.74	-3.31	1.74E-04	1.90E-02	down	cytochrome c
MXAN_5799	1184.27	2664.99	-1.17	2.09E-04	2.18E-02	down	type IV secretion protein Rhs
MXAN_6263	437.17	1575.49	-1.85	2.70E-10	1.04E-07	down	lysine 2,3-aminomutase
MXAN_6550	118.56	346.32	-1.55	8.78E-05	1.09E-02	down	glycoside hydrolase

							family 16 protein
MXAN_6551	197.07	496.79	-1.33	2.32E-04	2.39E-02	down	peptide ABC transporter substrate-binding protein
MXAN_6999	226.7	822.9	-1.86	8.45E-09	2.68E-06	down	TIGR02265 family protein
MXAN_2127	1971.08	8954.29	-2.18	5.94E-15	4.82E-12	down	hypothetical protein
MXAN_5033	5608.14	11890.49	-1.08	2.90E-05	3.93E-03	down	hypothetical protein
MXAN_6548	340.73	1105.96	-1.7	1.91E-04	2.05E-02	down	hypothetical protein
MXAN_6797	167.38	505.23	-1.59	3.99E-06	6.10E-04	down	hypothetical protein
MXAN_2124	18.93	87.45	-2.21	3.14E-04	3.10E-02	down	hypothetical protein

Footnotes: (1) Named MXAN_RS06635 in GenBank NC_008095.1; (2) Named MXAN_RS19940 in GenBank NC_008095.1

Table S4. Sequenced myxobacteria genome size and RecA duplication.

Suborder	Family	Genus	Sequenced strains	Genome size (bps）	Number of RecA
Cystobacterineae	Cystobacteraceae	1.Cystobacter	Cystobacter fuscus	12349744	2
		2.Hyalangium			
		3.Archangium	Archangium gephyra	12489432	2
		4.Stigmatella	Stigmatella aurantiaca	10260756	2
		5.Melittangium	Melittangium boletus	9910441	2
		6.Anaeromyxobacter	Anaeromyxobacter dehalogenans 2CP-C	5013479	1
			Anaeromyxobacter dehalogenans 2CP-1	5029329	1
			Anaeromyxobacter sp. Fw109-5	5277990	1
			Anaeromyxobacter sp. K	5061632	1
	Myxococcaceae	7.Myxococcus	Myxococcus xanthus	9139763	2
			Myxococcus fulvus	9003593	2
			Myxococcus stipitatus	10350586	2
			Myxococcus hansupus	9490432	2
			Myxococcus macrosporus	8973512	2
		8.Corallococcus	Corallococcus coralloides	10080619	
		9.Pyxicoccus			
Sorangineae	Polyangiaceae	10.Ployangium			
		11.Chondromyces	Chondromyces crocatus	11388132	2
		12.Sorangium	Sorangium cellulosum So ce56	13033779	2
			Sorangium cellulosum So0157-2	14782125	2
		13.Byssovorax			
		14.Jahnella			
		15.Haploangium			
	Phaselicystidaceae	16.Phaselicystis			
	Sandaracinaceae	17.Sandaracinus	Sandaracinus amylolyticus	10327335	2
Nannocystineae Suborder	Nannocystaceae	18.Nannocystis			
		19.Enhygromyxa			
		20.Plesiocystis			
		21.Pseudenhygromyxa			
	Haliangiaceae	22.Haliangium	Haliangium ochraceum	9446314	2
	Kofleriaceae	23.Kofleria			
