# Supplementary material for: Functional Division Between the RecA1 and RecA2 Proteins in Myxococcus xanthus
Source: Front Microbiol. 2020 Feb 12;11:140. doi: 10.3389/fmicb.2020.00140 (PMC7029660; doi:10.3389/fmicb.2020.00140)
Supplement: Supplementary file 2 [file Data_Sheet_2.pdf]

Figure S1

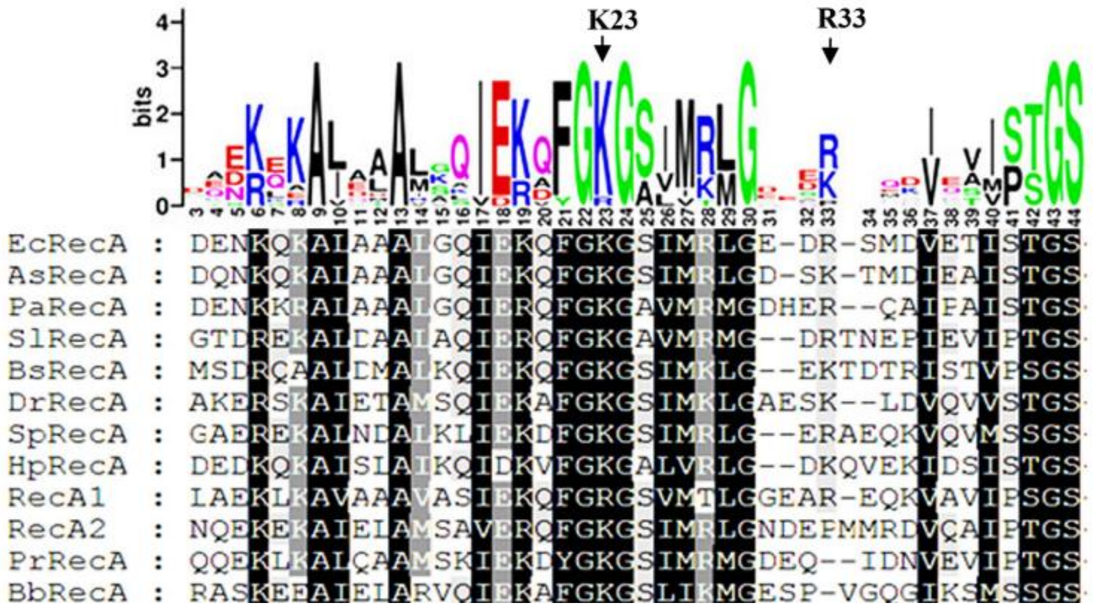

Figure S1. Amino acid sequence alignment of N-terminal domain of RecAs from different bacteria, including EcRecA from *E. coli* (b2699) (Cox, 1999), AsRecA from *Aeromonas salmonicida* (ASA_3809) (Umelo et al., 1996), PaRecA from *Pseudomonas aeruginosa* (PA3617) (Sano et al., 1987), SlRecA from *Streptomyces lividans* (SLIV_09770) (Nussbaumer et al., 1994), BsRecA from *Bacillus subtilis* (BSU16940) (Carrasco et al., 2019), DrRecA from *Deinococcus radiodurans* (DR_2340) (Kim et al., 2002), SpRecA from *Streptococcus pneumoniae* (SP_1940) (Grove et al., 2012), HpRecA from *Helicobacter pylori* (HP0153) (Orillard et al., 2011), PrRecA from *Prevotella ruminicola* (PRU_0066) (Aminov et al., 1996) and BbRecA from *Borrelia burgdorferi* (BB_0131) (Huang et al., 2017). Two key amino acids are noted (black arrow), and numbering is shown based on that of *E. coli* RecA.

Figure S2

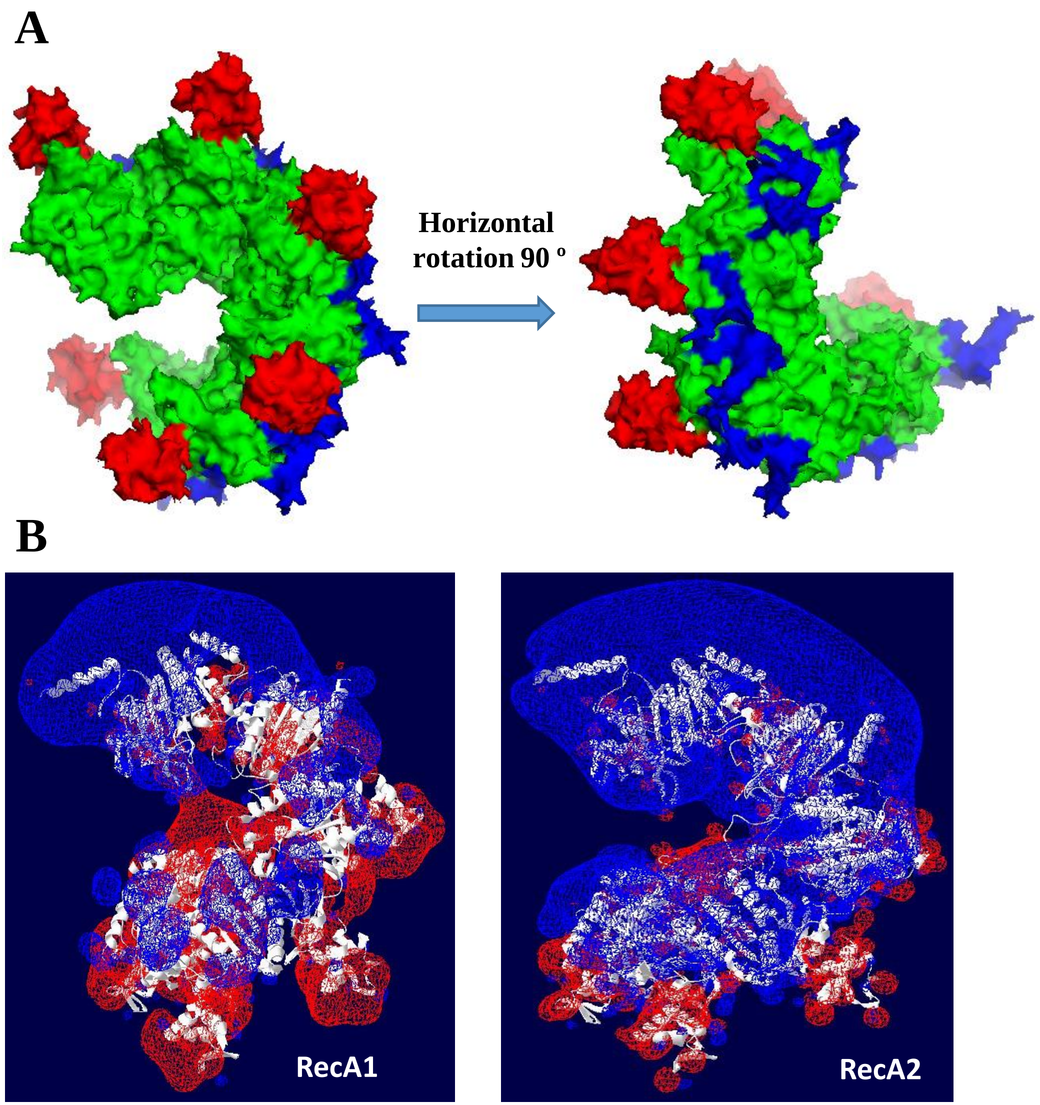

Figure S2. Surface charge analysis of RecA1 and RecA2. (A) RecA polymer model. The N-terminal domain (NTD, colored red) and C-terminal domain (CTD, colored blue) of RecA are both in the outer side, and core ATPase domain (CAD, colored green) is in the inner side of the polymer. The right one is the left one flipping 90 degrees horizontally. (B) Surface charge of RecA1 and RecA2. Blue represents negative charge and red represents positive charge.

Figure S3

A

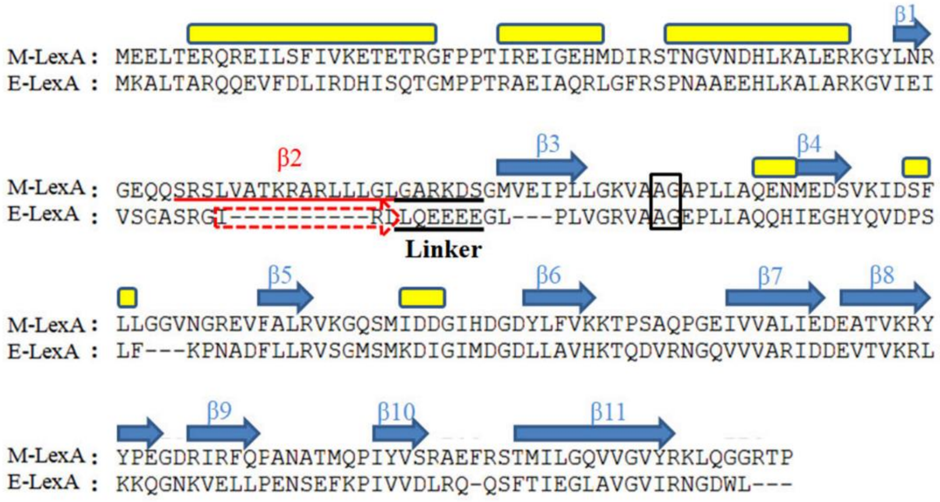

B

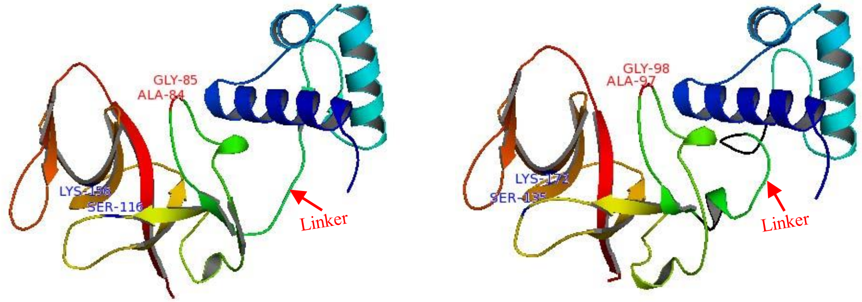

EcLexA (pI=6.23) **MxLexA (pI=8.77)**

EcLexA Linker: <u>LQEEEE</u> Theoretical pI: 3.58
MxLexA Linker: <u>SRSLVATK RARLLLGLGA RKDS</u> Theoretical pI: 12.01

Figure S3. Sequence and structural comparison of *M. xanthus* LexA (MxLexA) and *E. coli* LexA (EcLexA). (A) Sequence alignment of MxLexA and EcLexA using the MUSCLE program. The LexA self-cleavage site (A-G) of was marked in a black box. The linkers between the N- and C-terminal domains of the two LexAs are underlined. (B) Location of Linkers in protein structure. The 3D structure of MxLexA was constructed using homology modeling with EcLexA PDB structure (1JHF) as template. The linkers between the N- and C-terminal domains are indicted with red arrow. The sequences and theoretical pI values of the linkers of EcLexA and MxLexA are listed at the bottom of this figure.

Figure S4

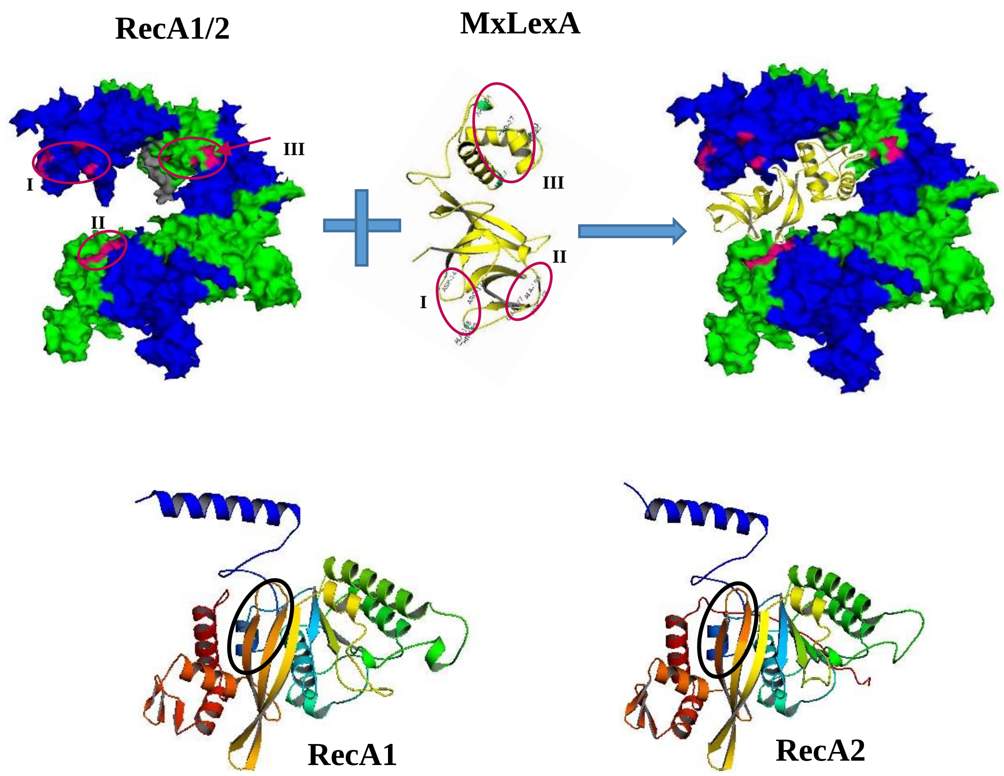

	Sequence (* above Q)	Theoretical pI
RecA1	VVKNKVAPPFQEAEF	6.11
RecA2	VVKNKVAPPFKEVEF	8.47

Figure S4. Simulated docking of LexA and RecA polymers. LexA and RecA bind to each other mainly through three binding regions (Kovačič et al., 2013), which are marked in red circle (upper map). The possible binding region of the Linker of LexA on RecA polymer is marked with grey and marked with black circles in RecA1 and RecA2 (lower map). The amino acid sequence of the linker binding regions and their corresponding pIs are listed below the figure.

Figure S5

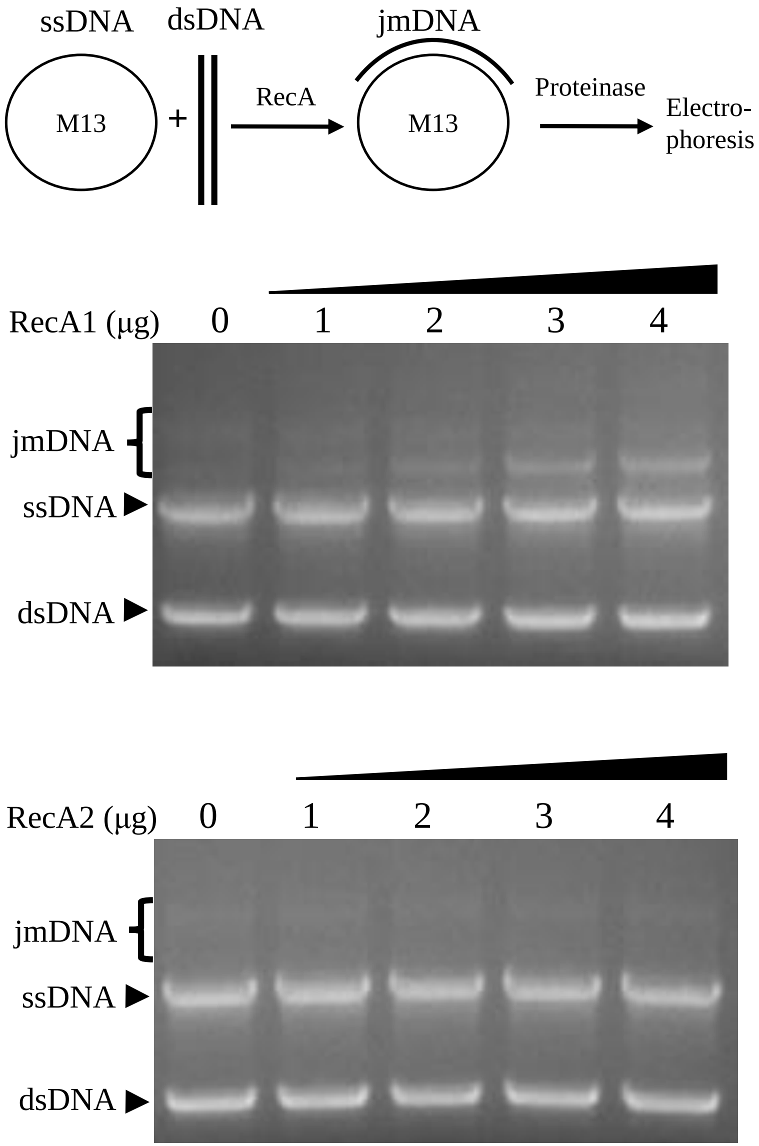

Figure S5. DNA strand exchange reaction promoted by RecA between M13 circular ssDNA and the linear dsDNA (derived from M13). Reactions were performed at 30 ° C in a solution containing 25 mM Tris-HCl, pH 7.0, 50 mM NaCl, 4% glycerol, 1 mM DTT, 10 mM $MgCl_2$, 3 mM ATP and an ATP-regenerating system (10 units/ml of pyruvate kinase/3.3 mM phosphoenolpyruvate). After pre-incubation of ssDNA with RecA1 or RecA2 protein at 30 ° C for 5 min, linear duplex DNA was added to start the DNA strand exchange reactions. The ssDNA and dsDNA substrates, as well as the joint molecule intermediates (jmDNA) bands are all visible in the 0.8% agarose gel.

Figure S6

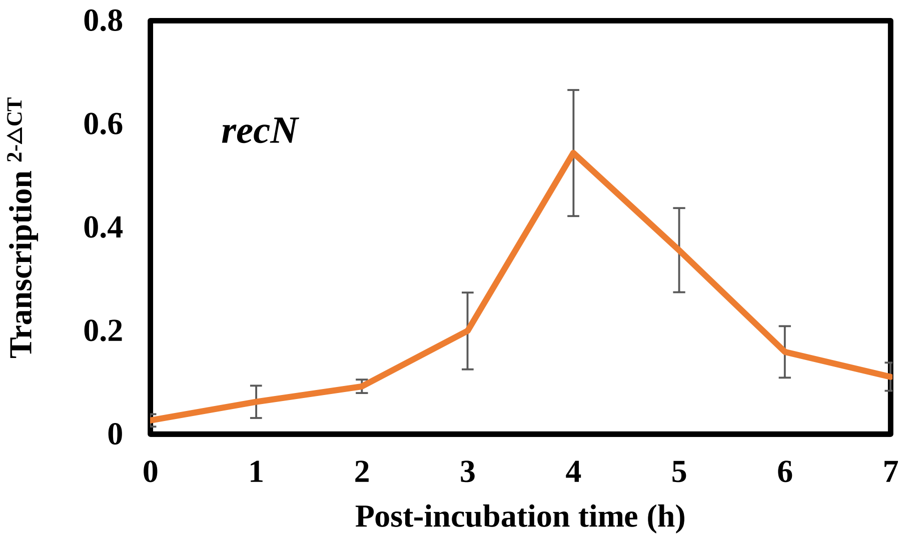

Figure S6. The induction time points of LexA-independent SOS gene *recN*. After exposed to UV irradiation at the dose of 15 J/m^2, the cell cultures were post-incubated at 30 °C, sampled at intervals to extract the total RNA for RT-PCR. The error bars represent means ± SEM (n=3).

Figure S7

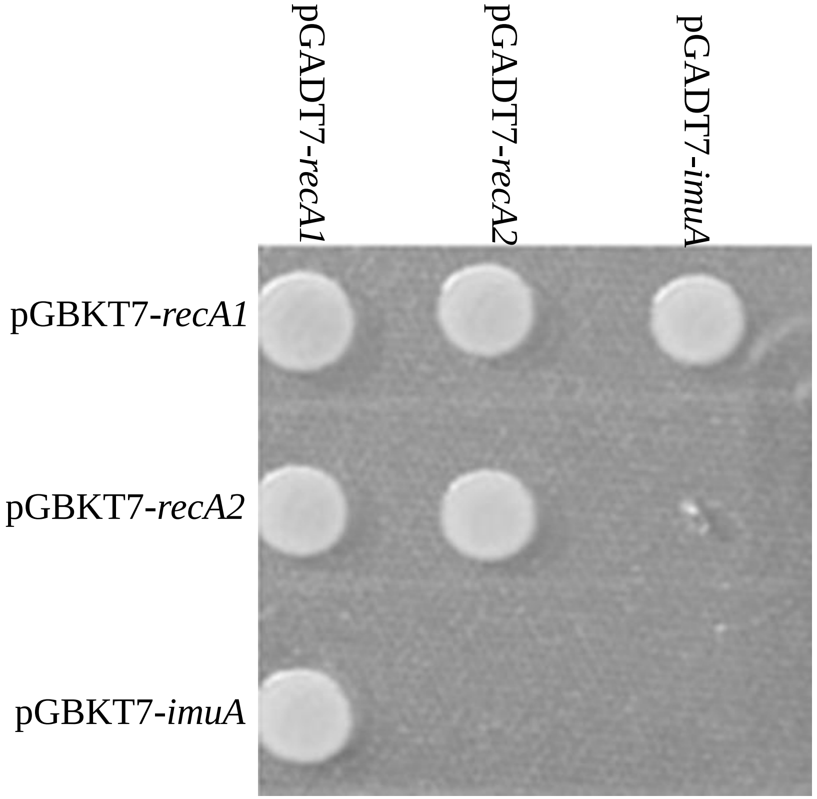

Figure S7. The yeast two hybrid (H2Y) analysis the interaction among with ImuA, RecA1 and RecA2. Genes *imuA*, *recA1* and *recA2* were amplified by PCR using primers ImuAF 5’-TACATATGAGCGCGGCGGAGCAGCGAGTG-3’/ImuAR 5’-TAGAATTCCGGGTGAGGTGCAGATAGG-3’, RecA1F 5’-CTCATATGGCCGTGAATCAGGAGAAGG-3’/RecA1R 5’-TTGAATTCGGACTACTTCACGGCCTTCACAC-3’ and RecA2F 5-TACATATGAGCAAGCTGGCGGAGAAG-3/RecA2R 5-AAGAATTCCGGTCAAGCTGGACGTGTT-3, respectively. The amplified DNA fragments of *imuA*, *recA1* and *recA2* were digested with *Nde* I and *Eco*RI, and then ligated into pGBKT7 (Clontech, CA, USA) to obtain pGBKT7-*imuA*, pGBKT7-*recA1* and pGBKT7-*recA2*, respectively. Similarly, the three genes were ligated into pGADT7 (Clontech) to generate pGADT7-*imuA*, pGADT7-*recA1* and pGADT7-*recA2*. Three pGBKT7 recombinant plasmids and three pGADT7 recombinant plasmids were combined and co-transformed into yeast strain Y2HGold with the selection of transformants on SD/-Trp/-Leu agar plates. Transformants were spoted on SD medium without adenine, leucine, and tryptophan. All experiments were repeated at least three times.
